# Developing a Protocol for Management of Euglycemic Diabetic Ketoacidosis

**DOI:** 10.1007/s11892-025-01604-3

**Published:** 2025-09-03

**Authors:** Zulma Cardona, Jared G. Friedman, Nevin Kamal, Diana J. Oakes, Amisha Wallia, Grazia Aleppo, Sophia Brown, Courtney T. Makowski, Kyle Ritter, Emily D. Szmuilowicz

**Affiliations:** 1 Division of Endocrinology, Metabolism, and Molecular Medicine, Department of Medicine, Northwestern University Feinberg School of Medicine, 645 N Michigan Ave, Suite 530, Chicago, IL 60611 USA; 2 Northwestern Memorial Hospital, Chicago, IL USA

**Keywords:** Euglycemic diabetic ketoacidosis, SGLT2 inhibitor, DKA, Hospital protocol, Insulin infusion

## Abstract

**Purpose of Review:**

Euglycemic diabetic ketoacidosis (euDKA) has been described since the 1970s, however the incidence appears to be increasing in association with the increased use of sodium-glucose cotransporter 2 inhibitor (SGLT2i) medications. Traditional hospital-based DKA protocols in which an insulin infusion is adjusted based on glucose levels are not effective in euDKA due to the presence of euglycemia which limits the capacity for insulin administration. This review was completed to review the data on euDKA and introduce a protocol for targeted management of this condition.

**Recent Findings:**

Data comparing euDKA outcomes to traditional hyperglycemia DKA demonstrate longer hospital length of stay and mean time to anion gap closure in euDKA based on current DKA management standards. Furthermore, the increase in prescribing SGLT2i medications thereby increases the risk of euDKA. At present, there are no reported protocols specific for euDKA and it is not directly addressed in the most recent guidelines issued by Endocrinology specialty societies.

**Summary:**

We created a protocol within our hospital intensive care unit to standardize treatment of euDKA using fixed insulin infusion and titration of dextrose-containing fluids. The protocol has been approved by our hospital regulatory committees and is currently being utilized in intensive care units. Future studies should review ongoing safety and efficacy of protocol use in various hospital settings.

## Introduction

In the realm of diabetes management, sodium-glucose cotransporter 2 inhibitor (SGLT2-i) medications are newer agents that have gained prominence due to their significant cardiovascular and renal protective effects [1, 2]. With increasing use of this class of medications, associations have emerged between SGLT2-i use and euglycemic diabetic ketoacidosis (euDKA) – a phenomenon whereby ketoacidosis develops in the setting of normal or slightly elevated glucose levels [3]. Clinical trials and meta-analyses, have demonstrated a higher incidence of euDKA in individuals with type 1 diabetes using SGLT2-i compared to those on placebo [4]. Though less frequent, an elevated risk of euDKA has also been observed in individuals with type 2 diabetes taking these agents compared to placebo [5].

### Diagnosis of euDKA and DKA

Current diagnostic criteria for typical DKA include the triad of glucose ≥ 200 mg/dL (or prior history of diabetes), ketosis, and metabolic acidosis [6]. In euDKA however, blood glucose levels are near-normal or normal and generally less than 200 mg/dL (sometimes defined as up to 300 mg/dL) [3]. Given the absence of hyperglycemia, osmotic symptoms such as polyuria, polydipsia, and other findings of hypovolemia may be absent in euDKA [7]. Additionally, symptoms in euDKA develop more gradually compared to the conventional hyperglycemic DKA, and with the lack of hyperglycemia and associated osmotic symptoms, diagnosis can be delayed or sometimes missed [7]. The pathophysiology of euDKA remains poorly understood. Known risk factors include very-low-carbohydrate diets or starvation, dehydration, excessive alcohol intake, and the presence of autoimmunity, as well as impaired gluconeogenesis from either chronic liver disease or alcohol use, cocaine use, pancreatitis, and sepsis [7, 8]. Additional risk factors include trauma, stroke, surgery, and prolonged exercise [9]. Individuals on insulin therapy who significantly reduce their insulin dosage in the setting of illness are also at increased risk for developing euDKA due to relative insulin deficiency [10]. Pregnancy is also a known risk factor for development of euDKA as it is a ketosis-prone state [11]. A high clinical suspicion for euDKA is needed for an accurate diagnosis and timely management.

### SGLT2-i Therapy and DKA

The mechanism of action of SGLT2-i is a major contributor to the development of euDKA. SGLT2-i medications directly act on SGLT2 receptors in the proximal convoluted tubules in nephrons, thereby inhibiting renal glucose re-absorption, inducing glycosuria and natriuresis and resulting in volume depletion and lower blood glucose levels. This leads to insufficient endogenous production of insulin, which in the setting of elevated levels of counter-regulatory hormones such as cortisol, epinephrine and glucagon, leads to fatty acid metabolism and ketone production despite near-normal blood glucose levels [3, 12].

When compared to conventional hyperglycemic DKA, SGLT2-i associated euDKA is associated with worse outcomes. One center demonstrated a mean time to anion gap closure of 17 h with SGLT2-i associated euDKA compared to 9.8 h with other DKA admissions at the same hospital, and the former also had significantly increased hospital length of stay [13]. Another study noted similar results when comparing euDKA to hyperglycemic DKA and attributed the prolonged time to anion gap closure to lower insulin infusion rates, presumably chosen to prevent hypoglycemia [14]. Effects of SGLT2-i may last up to 4–5 half-lives following administration (48–72 h), which contributes to the prolonged time to anion gap closure compared to conventional DKA [15].

While SGLT2-i were initially approved for a primary indication of diabetes, landmark trials followed (including EMPEROR-Reduced and EMPEROR-Preserved) that demonstrated significant cardiovascular benefits regardless of the presence or absence of diabetes in individuals with heart failure with reduced as well as preserved ejection fraction [16, 17]. This has led to marked increases in SGLT2-i prescribing rates for individuals with heart failure with reduced ejection fraction from 4% in 2021 to 24% in 2023 in one multi-site study of US hospitals [18]. Additionally, the EMPA-KIDNEY trial demonstrated reduced risk of progression of kidney disease in individuals with chronic kidney disease with use of SGLT2-i which ultimately led to FDA approval for use in chronic kidney disease regardless of diabetes status [19]. A meta-analysis of 39 RCTs involving over 60,000 individuals treated with SGLT2-i medications estimated a DKA prevalence of 0.18% [5]. With the significant rise in SGLT2-i prescriptions in recent years – from 700,000 prescriptions nationally in 2015 to 1.4 million in 2020 – the prevalence of SGLT2-i associated euDKA is likely to increase significantly [20].

To date, the most recent guidelines issued by the American Diabetes Association (2025), the American Association of Clinical Endocrinologists and American College of Endocrinology (2020) do not provide specific guidance regarding management of euDKA [8, 21]. A meta-analysis of 39 RCTs involving over 60,000 individuals treated with SGLT2-i medications estimated a DKA prevalence of 0.18% [5].Conventional hyperglycemic DKA can be treated with either a fixed-rate intravenous (IV) insulin infusion or by a variable-rate infusion protocol depending on institutional resources and DKA severity [6, 22]. Variable-rate insulin infusion protocols used to treat conventional hyperglycemic DKA, in which the insulin infusion rate is adjusted according to glucose levels, should not be used to treat euDKA [8]. In euDKA, treatment requires a fixed-rate IV insulin infusion to treat the ketoacidosis irrespective of the presenting glucose level, and dextrose-containing IV fluids must be titrated to maintain euglycemia while the fixed-rate insulin infusion needed to treat the ketoacidosis is infused.

A PubMed search was conducted using the terms “euglycemic diabetic ketoacidosis”, “management”, and “protocol” for articles published between 2020 and 2025. A total of 13 sources were reviewed and 7 were deemed relevant to the focus of this manuscript (sources that were not directly related to inpatient management of euDKA or SGLT2-i exposure were excluded). A retrospective review of patients with hyperglycemic DKA compared to patients with euDKA who were treated with a fixed insulin infusion protocol with titratable dextrose reported that euDKA patients had shorter mean time on insulin infusion but hypoglycemia incidence was higher compared to the hyperglycemic DKA group. Details on the protocol were not provided in the publication [23]. A number of case reports linking euDKA with SGLT2-i have been published with various individual management strategies detailed [24–28]. Additionally, a review of case reports of SGLT2-i associated euDKA in patients concurrently infected with coronavirus disease 2019 (COVID-19) were managed with a conventional DKA protocol and highlighted the need for standardized protocol for management [29].

While administration of a fixed infused infusion rate is central to treatment of euDKA there are scant recommendations in the existing literature and most recent guidelines regarding protocolized adjustment of dextrose-containing IV fluids and monitoring [6, 30, 31]. This highlights a critical gap in the existing literature, which lacks specific guidance on fluid rate adjustments in the management of euDKA. Timely diagnosis and treatment in euDKA is critical and without a standardized, reliable and replicable protocol, individuals with euDKA may receive inconsistent management and face suboptimal outcomes. Our institution therefore developed a protocol to address this need.

## Protocol Description

This document outlines a protocol designed to standardize the treatment of euDKA within emergency and intensive care settings. The protocol provides comprehensive guidelines for the management of intravenous regular human insulin (RHI) infusion, fluid therapy, electrolyte repletion, and laboratory monitoring.

### Indications for Initiation

The protocol is initiated by the primary service upon diagnosing euDKA. They initiate the process by utilizing an order set that is currently available in our institutional electronic health record. In our protocol, euDKA is defined by a glucose level < 250 mg/dL (our protocol was developed prior to publication of the 2024 Consensus Report noting that current guidelines now define it as glucose < 200 mg/dL [6]), accompanied by severe metabolic acidosis (pH < 7.3 and serum bicarbonate < 18 mEq/L), with the presence of serum and/or urine ketones (beta-hydroxybutyrate levels > 3 mmol/L). An anion gap > 12, may further support the diagnosis [6, 32, 33].

When the above criteria are met, initial laboratory evaluations should ideally include a STAT basic chemistry panel, arterial blood gas (ABG) or venous blood gas (VBG). While an ABG is generally considered the gold standard for assessing acid-base balance, in the context of DKA, a VBG is often considered a suitable alternative because the difference in pH between arterial and venous blood is usually clinically insignificant, making VBG less invasive and more patient-friendly [34]. Additionally, a urinalysis, lactic acid, and beta-hydroxybutyrate levels should be obtained.

**Box 1:** Consultation with the endocrinology service, and/or evaluation should be considered in the following cases

• Persistent anion gap elevation (> 12 mmol/L) after two attempts to transition from intravenous (IV) to subcutaneous insulin therapy	
• End Stage Renal Disease (ESRD)	
• Type 1 Diabetes Mellitus (T1D)	

### Titration and Monitoring

Management strategies include hydration, starting with an initial fluid bolus followed by maintenance intravenous fluids, such as normal saline or Lactated Ringer’s solution, tailored to the individual’s clinical needs. A dextrose 5% (D5) infusion at 150 mL/hour or dextrose 10% (D10) at 75 mL/hour is administered, with D10 used for individuals at risk of volume overload or persistent hypoglycemia. The dextrose-containing fluids are adjusted based on point-of-care glucose measurements to maintain a glucose goal of 140–180 mg/dL [see Tables 1 and 2], enabling continuous administration of the fixed-rate insulin infusion necessary to treat the ketoacidosis. Insulin infusion is initiated without an initial bolus and maintained at a fixed rate of 0.1 unit/kg/hour to support ketone body clearance and resolution of acidosis. For individuals ≥ 150 kg, the starting insulin infusion rate is 0.05 unit/kg/hour to mitigate risk of excess insulin dosing. Point-of-care glucose is monitored hourly throughout the protocol, with titration adjustments as depicted in Tables 1 and 2.

**Table 1:** Titrations of dextrose-containing fluids for blood glucose > 140 mg/dl

CURRENT BLOOD GLUCOSE (BG)	Insulin drip rate ** For patients ≥ 150 kg, insulin rate will be 0.05 units/kg/hr as above ** ** NO TITRATION **	Maintenance IV fluid rate (LR or NS)	Dextrose Infusion Rate			
**200–250 mg/dL**	0.1 u/kg/hr	83 mL/hr, or as dictated by provider for volume status	**BG decreased** **by < 50 mg/dL**	**BG decreased** **by > 50 mg/dL**	**BG increased** **by < 50 mg/dL**	**BG increased** **by > 50 mg/dL**
			For dextrose **5%**: Decrease D5 infusion by 50 ml/hr.	For dextrose **5%**: Decrease D5 infusion by 25 ml/hr.	For dextrose **5%**: Decrease D5 infusion by 75 ml/hr.	For dextrose **5%**: Decrease D5 infusion by 100 ml/hr.
			For dextrose **10%**: Decrease D10 infusion by 25 ml/hr.	For dextrose **10%**: Decrease D10 infusion by 12.5 ml/hr.	For dextrose **10%**: Decrease D10 infusion by 37.5 ml/hr.	For dextrose **10%**: Decrease D10 infusion by 50 ml/hr.
**180–199 mg/dL**	0.1 u/kg/hr	83 mL/hr, or as dictated by provider for volume status	**BG decreased** **by < 50 mg/dL**	**BG decreased** **by > 50 mg/dL**	**BG increased** **by < 50 mg/dL**	**BG increased** **by > 50 mg/dL**
			For dextrose **5%**: Decrease D5 infusion 25 ml/hr. For dextrose **10%**: Decrease D10 infusion by 12.5 ml/hr.	Keep dextrose-containing fluid at current rate. Repeat blood glucose check in 1 h and follow the instructions below: --If repeat BG decreases by *> 25 mg/dL*: Keep dextrose infusion at current rate. --If repeat BG is *still between 180–199 mg/dL*: For dextrose **5%**: Decrease D5 infusion by 25 ml/hr. For dextrose **10%**: Decrease D10 infusion by 12.5 ml/hr. --If repeat BG *decreases by 0–24 mg/dL and is NOT within 180–199 mg/dL*: Adjust dextrose based on recommended rate for new blood glucose.	For dextrose **5%**: Decrease D5 infusion by 50 ml/hr.	For dextrose **5%**: Decrease D5 infusion by 75 ml/hr.
					For dextrose **10%**: Decrease D10 infusion by 25 ml/hr.	For dextrose **10%**: Decrease D10 infusion by 37.5 ml/hr.
**140–179 mg/dL**	0.1 u/kg/hr	83 mL/hr, or dictated by provider for volume status	Continue dextrose-containing infusion at current rate.			

*Based on first Blood Glucose result 1 h after initiating dextrose infusion*

**Table 2:** Titrations of dextrose-containing fluids for blood glucose < 140 mg/dl

CURRENT BLOOD GLUCOSE (BG)	Insulin drip rate * For patients ≥ 150 kg, insulin rate will be 0.05 units/kg/hr as above ** ** NO TITRATION **		Maintenance IV fluid rate (LR or NS)		Dextrose Infusion Rate						
**120–139 mg/dL**	0.1 u/kg/hr		83 mL/hr, or as dictated by provider for volume status		**BG decreased** **by < 50 mg/dL**		**BG decreased** **by > 50 mg/dL**		**BG increased** **by < 50 mg/dL**		**BG increased** **by > 50 mg/dL**
					For dextrose **5%**: Increase D5 infusion by 50 mL/hr.		For dextrose **5%**: Increase D5 infusion by 75 mL/hr.		For dextrose **5%**: Increase D5 infusion by 50 mL/hr.		For dextrose **5%**: Increase D5 infusion by 25 ml/hr.
					For dextrose **10%**: Increase D10 infusion by 25 mL/hr.		For dextrose **10%**: Increase D10 infusion by 37.5 mL/hr.		For dextrose **10%**: Increase D10 infusion by 25 mL/hr.		For dextrose **10%**: Increase D5 infusion by 12.5 mL/hr.
**80–119 mg/dL**	0.1 u/kg/hr		83 mL/hr, or as dictated by provider for volume status		For dextrose **5%**: Increase D5 infusion by 75 mL/hr.		For dextrose **5%**: Increase D5 infusion by 100 mL/hr.		For dextrose **5%**: Increase D5 infusion by 75 mL/hr.		For dextrose **5%**: Increase D5 infusion by 50 mL/hr.
					For dextrose **10%**: Increase D10 infusion by 37.5 mL/hr.		For dextrose **10%**: Increase D10 infusion by 50 mL/hr.		For dextrose **10%**: Increase D10 infusion by 37.5 mL/hr.		For dextrose **10%**: Increase D10 infusion by 25 mL/hr.
**70–79 mg/dL**		0.1 u/kg/hr		83 mL/hr, or as dictated by provider for volume status		**BG decreased** **by < 50 mg/dL**		**BG decreased** **by > 50 mg/dL**		**BG increased** **by < 50 mg/dL**	**BG increased** **by > 50 mg/dL**
						Give 25 g dextrose as IV push or 250 mL IV bolus of 10% dextrose over 15 min, and page primary service. For dextrose **5%**: Increase D5 infusion by 100 mL/hr.		Give 25 g dextrose as IV push or 250 mL IV bolus of 10% dextrose over 15 min, and page primary service. For dextrose **5%**: Increase D5 infusion by 125 mL/hr.		Give 25 g dextrose as IV push or 250 mL IV bolus of 10% dextrose over 15 min, and page primary service. For dextrose **5%**: Increase D5 infusion by 100 mL/hr.	Give 25 g dextrose as IV push or 250 mL IV bolus of 10% dextrose over 15 min, and page primary service. For dextrose **5%**: Increase D5 infusion by 75 mL/hr.
						For dextrose **10%**: Increase D10 infusion by 50 mL/hr.		For dextrose **10%**: Increase D10 infusion by 62.5 mL/hr.		For dextrose **10%**: Increase D10 infusion by 50 mL/hr.	For dextrose **10%**: Increase D10 infusion by 37.5 mL/hr.
						Re-check BG in 1 h. Adjust dextrose infusion rate to recommended rate based on repeat BG level change.					

*Based on first Blood Glucose result 1 h after initiating dextrose infusion*

** If blood glucose falls below 70 mg/dL: Hold insulin infusion for 1 h and page primary service. Give 25 g dextrose as IV push or 250 mL IV bolus of 10% dextrose over 15 min

1. If dextrose 5% running; increase D5 infusion by 125 ml/hr

2. If dextrose 10% infusion running: increase D10 infusion by 62.5 mL/hr

3. Repeat BG in 15 min and if 70 mg/dL, repeat treatment with 25 g dextrose. Check BG every 15 min until it reaches >70 mg/dl with dextrose repletion. After 1 h, resume insulin drip at previous set rate and continue to adjust dextrose infusion to recommended rate based on above chart

4. If persistent hypoglycemic episodes occur despite high rates of dextrose infusion, page covering provider AND pharmacist to discuss decreasing set insulin infusion rate to 0.05 units/kg/hr

For initial serum potassium levels *≤* 4.4 mEq/L, potassium should be repleted to achieve a goal of 4.5–5.3 mEq/L prior to initiation of the insulin infusion. Insulin infusion should only be initiated concomitantly with dextrose-containing and maintenance fluids when potassium levels exceed 3.3 mEq/L.

**Box 2:** Laboratory Monitoring

• Blood glucose levels (point-of-care) every hour	
• Basic metabolic panels every 2 h.	

As noted in the protocol, if blood glucose falls below 70 mg/dL, the insulin infusion should be held for one hour and dextrose should be administered. If persistent hypoglycemic episodes (i.e. more than one consecutive event) despite high rates of dextrose infusion, then the set insulin infusion rate may be decreased in order to prevent risk of hypervolemia and hyponatremia secondary to dextrose 5% infusion rates greater than 250 mL/hr.

### Transitioning Intravenous Insulin Infusion To Subcutaneous Insulin

Ensure that the SGLT2-i has been eliminated before discontinuing insulin and dextrose infusions, as ketoacidosis may recur if insulin therapy is discontinued prematurely before SGLT2-i has been fully cleared. Depending on last administration of SGLT2-i, insulin infusion should be continued for at least 48 h or longer based on half-life elimination of the medication of approximately 12 h. Of note, this elimination time can be prolonged in the setting of altered renal function.

For insulin-naïve individuals, initiate subcutaneous long-acting basal insulin at 0.2 units/kg daily along with supplemental insulin per a correction scale for glucose values > 150 mg/dL. For individuals previously on insulin therapy, consult pharmacy or endocrinology for appropriate dose adjustments. Ongoing need for basal insulin and insulin dose adjustments should be made in response to glucose levels after transition to a subcutaneous insulin regimen.

## Discussion

Successful management of euDKA is challenging since a sufficient insulin dose must be given to allow for adequate clearance of ketones while dextrose-containing fluids must be concurrently administered at appropriate rates to prevent hypoglycemia. Even as far back as the first published description of euDKA in 1973, it had been reported that the proposed treatment would require large doses of insulin which would then in turn need to be “covered” by adequate carbohydrate such as 10% dextrose [35]. Provoking etiologies of euDKA other than SGLT2-i use can be wide ranging, but the onset usually occurs in the setting of underlying relative or absolute starvation such as fasting, alcohol, or pregnancy often combined with reduced insulin doses [36]. A review of the FDA Adverse Event Reporting System suggested that SGLT2-i exposure was linked to a seven-fold increase of developing acidosis in individuals with type 2 diabetes [37]. It was previously estimated that even before FDA approval of SGLT2-i medications, approximately 2.6% of individuals with DKA had euDKA and thus incidence rates would be expected to increase with higher prescribing and utilization of this class of medication [38]. As SGLT2-i medications continue to show promising benefits for improving clinical outcomes for individuals without diabetes who have heart failure with reduced ejection fraction, heart failure with preserved ejection fraction, and chronic kidney disease, prescription of SGLT2-i medications has increased substantially for these indications, including a 12-fold increase in monthly prescribing by cardiologists between 2015 and 2020 [20, 39, 40]. Even individuals without diabetes who have intact insulin production have been demonstrated to be at risk for euDKA when triggered by stress and reduced oral intake [41].

To prevent euDKA, patient education is crucial, particularly regarding ‘sick day rules.’ Patients should be instructed to temporarily stop the medication if they experience symptoms such as nausea or vomiting and to monitor both blood glucose and ketone levels frequently during illness [3]. Ketone monitoring can be carried out via urine test strips or a blood ketone meter. If ketones are trace/small or 0.6–1.5 mmol/L it is advised that patients ingest 15–30 g of carbohydrate, administer a corresponding dose of rapid acting insulin, maintain fluid ingestion of 300–500 mL hourly (although recommended volume varies based on cardiac and renal status), and monitor ketones every three to four hours until normalization [33]. These recommendations can also be followed for moderate ketones in urine or blood ketones 1.6–3.0 mmol/L although medical attention is recommended; if urine ketones are large or blood ketones are > 3.0 mmol/L then immediate medical attention should be sought [33]. It is important to emphasize that normal blood glucose levels do not rule out the possibility of euDKA. Expert recommendations also advise discontinuing SGLT2-i for three days before surgery (with the exception of ertuglifozin which should be held for four days) to reduce the risk of postoperative euDKA [9, 42]. However, recent data has suggested that preoperative use of SGLT2-i in patients that underwent emergency surgery was not associated with an increased risk of euDKA so guidance on withholding these medications prior to surgery may evolve, especially if the medication is being used for a cardiac or renal indication [43]. If an identifiable and avoidable trigger for DKA is found, resuming the medication may be considered in some cases; however, in general, patients who develop euDKA while on therapy are advised to discontinue SGLT2-i altogether [3].

In the setting of anticipated increases in euDKA prevalence, we believe it is imperative to have a protocol in place to safely and effectively manage this serious adverse effect. Based on existing literature and our collective clinical experience we believe that a protocol in which the insulin infusion rate is fixed, and dextrose-containing fluids are titrated based upon hourly point-of-care blood glucose values allow for safe and effective resolution of euDKA.

This led to the development of our institutional protocol which was conceived in a multidisciplinary fashion with close collaboration between Endocrinology, Pharmacy, Nursing and Critical Care teams. As noted, the protocol clearly and specifically describes adjustments to dextrose-containing fluid rates based on glucose value and rate of glucose change as well as guidance for transitioning to subcutaneous insulin therapy and discussion of various instances in which the Endocrinology consult service is recommended to provide specialized assistance in management. For instances in which Endocrinology would be consulted but there is no Endocrinology service available, then consultation with the service that would otherwise be responsible for management of complex DKA is recommended. If unavailable or unable to provide this level of care, then transfer to a tertiary care center should be considered.

Initially, the Pharmacy group completed a pilot Quality Improvement project reviewing protocol use and various endpoints including length of stay in the intensive care unit and euDKA recurrence as well as safety endpoints such as hypoglycemia episodes. Initial experiences with implementation of this protocol at our institution indicate safety and efficacy; formal systematic evaluation of safety and efficacy is forthcoming and is the subject of ongoing work. The protocol was approved by our institutional Glycemic Control Committee and was integrated into our electronic health record platform as an “order set” and released for clinical use in April 2024. To our knowledge, this is the first reported protocol for euDKA management using a titration scheme of dextrose-containing fluids (as seen in Table 1) as well as guidance on appropriate lab monitoring. We hope that by sharing this protocol with other clinical sites we can facilitate a standardized management strategy for euDKA which should be applicable in various hospital settings with different levels of resources. Moving forward, we hope to collect prospective data to demonstrate the ongoing safety and efficacy of the protocol.

## Conclusion

EuDKA is an increasingly recognized entity associated with SGLT2-i use for which there are not presently any formal guidelines for management. Our institution developed a protocol for use of a fixed-rate insulin infusion with titration of continuous dextrose-containing IV fluids based upon blood glucose and glycemic trends. This protocol has been formally approved by our hospital regulatory committees and is now being utilized in intensive care units throughout our hospital system for the management of euDKA.

## Key References

- Impact of diabetes on the effects of sodium glucose co-transporter-2 inhibitors on kidney outcomes: collaborative meta-analysis of large placebo-controlled trials. Lancet. 2022;400(10365):1788 − 801. doi: 10.1016/s0140-6736(22)02074-8.
-
  - A large systematic review and meta-analysis of SGLT2-i trials that demonstrated data to support renal benefits of SGLT2-i use in patients with chronic kidney disease or heart failure without diabetes. This added to prior data supporting the use of SGLT2-i for cardiovascular benefits in patients with heart failure without diabetes.
- Chow E, Clement S, Garg R. Euglycemic diabetic ketoacidosis in the era of SGLT-2 inhibitors. BMJ Open Diabetes Res Care. 2023;11(5). doi: 10.1136/bmjdrc-2023-003666.
-
  - A review of euDKA that proposes a classification system for severity of euDKA and discusses an algorithmic approach to management of euglycemic DKA including recommended use of continuous dextrose-containing fluids—an approach that was incorporated into a formal titration scheme in our hospital protocol.
- Umpierrez GE, Davis GM, ElSayed NA, et al. Hyperglycaemic crises in adults with diabetes: a consensus report. *Diabetologia*. 2024;67(8):1455–1479. 10.1007/s00125-024-06183-8.
-
  - A comprehensive consensus statement on hyperglycemic crises (DKA and hyperglycemic hyperosmolar state) with updates on diagnosis, prevention, and treatment of these conditions.

## Data Availability

No datasets were generated or analysed during the current study.
